# Digital versus Analog Procedures for the Prosthetic Restoration of Single Implants: A Randomized Controlled Trial with 1 Year of Follow-Up

**DOI:** 10.1155/2018/5325032

**Published:** 2018-07-18

**Authors:** Francesco Mangano, Giovanni Veronesi

**Affiliations:** ^1^Department of Medicine and Surgery, Dental School, University of Varese, Italy; ^2^Department of Medicine and Surgery, Research Center in Epidemiology and Preventive Medicine, University of Varese, Italy

## Abstract

**Aim:**

To compare the outcome of digital versus analog procedures for the restoration of single implants.

**Methods:**

Over a two-year period (2014-2016), all patients who had been treated in a dental center with a single implant were randomly assigned to receive either a monolithic zirconia crown, fabricated with digital workflow (*test* group), or a metal-ceramic crown, fabricated with analog workflow (*control *group). All patients were followed for 1 year after the delivery of the final crown. The outcomes were success, complications, peri-implant marginal bone loss (PIMBL), patient satisfaction, and time and cost of the treatment.

**Results:**

50 patients (22 males, 28 females; mean age 52.6±13.4 years) were randomly assigned to one of the groups (25 per group). Both workflows showed high success (92%) and low complication rate (8%). No significant differences were found in the mean PIMBL between* test* (0.39±0.29mm) and* control* (0.54±0.32mm) groups. Patients preferred digital impressions. Taking the impression took half the time in the* test* group (20±5min) than in the* contro*l (50±7min) group. When calculating active working time, workflow in the* test* group was more time-efficient than in the* control* group, for provisional (70±15min versus 340±37min) and final crowns (29±9min versus 260±26min). The digital procedure presented lower costs than the analog (€277.3 versus €392.2).

**Conclusions:**

No significant clinical or radiographic differences were found between digital and analog procedures; however, the digital workflow was preferred by patients; it reduced active treatment time and costs. The present study is registered in the ISRCTN (http://www.isrctn.com/ISRCTN36259164) with number 36259164.

## 1. Introduction

The world of dentistry is now experiencing a revolution, thanks to the rapid establishment of digital technologies [1, 2]. New acquisition devices (intraoral scanners [3], face scanners, and cone-beam computed tomography (CBCT) [4] allow the capture of three-dimensional (3D) images of patients, which are then processed in computer-assisted design/computer-assisted manufacturing (CAD/CAM) software [5]—this is in order to be able to design and then produce, through subtractive technologies (milling) or additive (3D printing) methods, prosthetic restorations [1, 2, 5–7], surgical templates [8], orthodontic aligners [9], and a whole series of other custom-made devices (such as implants [10] and custom-made bone grafts [11]).

In implant prosthodontics, digital technologies today allow one to capture an accurate impression of the implant with intraoral scanners and therefore with structured light or laser only, without having to use conventional impression trays and materials [2, 3, 5, 12]. This new procedure is absolutely pleasing to patients, because it can reduce discomfort and stress while in the dental chair [2, 3, 5, 13, 14]; moreover, it is appreciated by the clinicians, as, besides being a powerful marketing tool with patients, it simplifies the clinical procedures and allows one to communicate in a more efficient and dynamic way with the dental laboratory [2, 3, 13, 15]. The modern dental laboratory, once it has received the optical impression, can proceed to the CAD design of the implant abutment and prosthetic crown, and can subsequently produce them through CAM procedures, such as milling [2–6, 13–16]. These restorations, appropriately characterized, will be sent to the dentist for clinical application [2–6, 13–18].

The replacement of the single missing or failing tooth with a dental implant is today one of the most frequent indications of modern implantology, with high percentages of survival and success, as unequivocally reported by the literature [19–22].

Digital technologies seem to represent an ideal application in implant prosthodontics, especially in the replacement of the single element, as recently demonstrated [2, 5, 6, 15–18, 23]. The digital procedure consists of the acquisition of an optical impression of the implant, through the positioning of a scanbody, able to transfer the spatial position of the fixture into the CAD software [5, 6, 15–18, 23]. In the CAD, the dental technician replaces the mesh of the scanbody with the file contained in the implant library and thus design the restoration [2, 5, 6, 15–18, 23]. The restoration is directly milled from a monolithic block of lithium disilicate [16, 23–26] or zirconia [15, 17, 18, 27, 28] (without the need to pass through a physical model), then characterized, and then delivered to the dentist. This restoration can be directly screwed onto a prefabricated titanium abutment with a dedicated shape, or it can be cemented on an individual hybrid abutment, the upper part of which is shaped and milled in zirconia and glued to a titanium bonding base [5, 6, 15, 17, 18, 27, 28].

The locking-taper, pure interference-fit implants (Morse taper connection implants, i.e., implants without a connecting screw between the abutment and the fixture) represent a successful long-term solution for prosthetic rehabilitation of single-tooth gaps [20, 29], as well as partially and totally edentulous jaws [30, 31], for supporting both fixed [20, 29–31] and removable prostheses [32]. In the case of single-tooth gaps, in particular, several clinical studies have shown that the use of such implants allows one to minimize the complications of a prosthetic nature [20, 29–31], particularly in the long term [29–31].

To date, there are very few randomized controlled studies in the literature that compare the procedures and results obtained in the replacement of the single teeth with digital versus analog (conventional) procedures [5, 15–18]; moreover, there are no studies that address this topic for implants with a Morse taper, pure interference-fit connection [5].

The aim of the present randomized clinical trial (RCT) was therefore to evaluate the success and complications encountered in the prosthetic restoration of single-tooth Morse taper connection implants, with digital and analog procedures, comparing the two methods; moreover, the present clinical trial aims to analyze and compare the patients' preference, the treatment times, and the costs, relative to the two different methodologies.

## 2. Materials and Methods

### 2.1. Patient Selection

Only patients who had undergone surgical treatment with the insertion of a single Morse taper connection implant (Exacone®, Leone Implants, Sesto Fiorentino, Italy) in the posterior areas (premolars and molars) of both jaws, in the period between September 2014 and September 2016, in a single dental center (Gravedona, Como, Italy), were considered for enrollment in the present RCT. A further inclusion criterion was the diameter and height of the implant received: the patients had to be installed with a fixture of a minimum diameter of 4.1 mm and a height of at least 8 mm. In order to be enrolled in the study, patients had to have dentition in the opposite jaw and therefore occlusal contacts. Finally, to be enrolled, patients had to read and sign a document of adhesion to the present study, on the nature (and possible therapeutic alternatives) of which they were informed in detail; by signing this document, they committed themselves to come to the dental clinic for the required follow-up appointments.

All patients who received a single implant with a diameter of less than 4.1 mm and a height of less than 8 mm were automatically excluded from this study, as were all patients who had undergone preimplant regenerative bone therapies or who had been treated with guided bone regeneration and membranes for the presence of peri-implant defects. Additional exclusion criteria included systemic diseases such as uncompensated diabetes, immunocompromised states, head and neck tumors, and osteoporosis treated with amino-bisphosphonates (administered orally and / or parenterally). Active periodontal infections and oral mucosa pathologies also represented exclusion criteria for enrollment in the present study. On the other hand, smoking and parafunctional habits (bruxism and/or clenching) did not represent exclusion criteria; smokers and bruxists could be included in this study.

The present RCT took place in full compliance with the principles set forth in the Helsinki Declaration on human subject experimentation of 1975 (and revision of 2008) and obtained the approval of the University of Insubria Ethics Committee, with approval number 826-0034086 (title: “Multicenter Clinical Studies on Survival and Success of Morse Taper Connection Implants”).

### 2.2. Study Design

The present study was designed as a randomized controlled clinical trial, for the comparison of two different prosthetic treatment modalities—digital versus analog procedures—for single implants inserted in the posterior areas of the jaws. Patients were therefore randomly assigned either to receive a monolithic zirconia crown, fabricated with a full digital workflow (*test* group: digital procedure consisting of digital impression with intraoral scanner, and CAD/CAM procedure without any physical model) or to receive a metal-ceramic crown, fabricated with a conventional analog workflow (*control* group: analog procedure consisting of impression-taking with polyvinyl siloxane, plaster model pouring, and lost-wax casting technique) (Table 1).

Block randomization was used in order to achieve a balanced allocation and an equal distribution of patients between the* test *and the* control* groups [33]. The randomization scheme consisted of a sequence of blocks, each block containing a prespecified number of treatment assignments, in random order [33].

The main outcomes of the study were clinical (implant-crown success and complications) and radiographic (peri-implant marginal bone loss), but patient satisfaction and time and costs of therapy in the two treatment groups were also compared.

**Figure 1:**
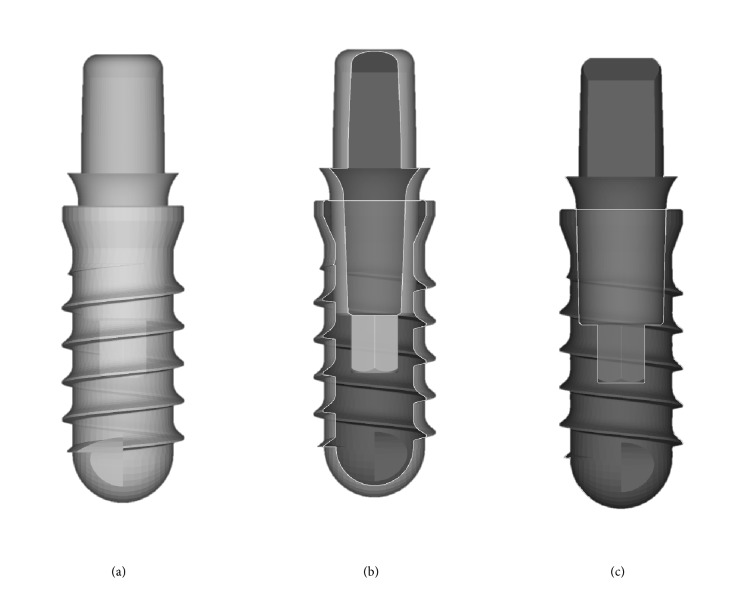
The fixtures used in the present study had a screwless Morse taper implant abutment connection (Exacone®, Leone Implants, Sesto Fiorentino, Italy) with a taper angle of 1.5°, combined with an internal hexagon. (a) Drawing of the fixture with the multitech abutment in position,* test* group; (b) section of the same assembly along the long axis, 30%; (c) section of the same assembly along the long axis, 50%.

In both groups of patients, the same Morse taper connection implants were used. This implant system (Exacone®, Leone Implants, Sesto Fiorentino, Italy) features a screwless, pure interference-fit connection between the fixture and the abutment, with an angle of 1.5°, combined with an internal hexagon [20, 29–31] (Figure 1).

The present randomized controlled trial has been registered in the ISRCTN publicly available register (http://www.isrctn.com/ISRCTN36259164) with number ISRCTN36259164.

### 2.3. Treatment Procedures

In both cases (either in the* test*—digital procedure; or in the* control*—analog procedure), the process started with the removal of the transmucosal healing abutment.

Digital treatment (*test* procedure) consisted of taking an optical impression with an intraoral scanner (CS 3600®, Carestream Dental, Rochester, NY, USA) after scanbody positioning. Scanning was limited to the posterior sector of interest, including the antagonist arch. This optical impression, in the form of  .STL or  .PLY files, was sent to the dental laboratory, which proceeded with the design in CAD software (Exocad DentalCAD®, Exocad, Darmstadt, Germany) of a definitive individual zirconia abutment to be cemented on a titanium base (link) and a temporary crown. The individual abutment was made of zirconia (for milling) with a powerful 5-axis milling machine (Roland DWX-50®, Roland Easy Shape, Ascoli Piceno, Italy), sintered in an oven (Tabeo®, Mihm-Vogt, Stutensee, Germany), and cemented extraorally on a straight (Ti-base® or Multitech straight®, Leone, Sesto Fiorentino, Italy) or 15°-angled (Multitech 15°®, Leone, Sesto Fiorentino, Italy) titanium base, selected according to the CAD project. The temporary crown, however, was produced by milling in polymethylmethacrylate (PMMA) with a 4-axis milling machine (Roland DWX-4®, Roland Easy Shape, Ascoli Piceno, Italy). Upon delivery of the temporary, the individual hybrid abutment was activated with a percussion hammer and the PMMA crown cemented on it with a zinc-oxide eugenol cement (TempBond®, Kerr, Orange, CA, USA). A careful occlusal check, polishing, and characterization was made. Any occlusal precontacts recorded in this phase with articulating papers (Bausch Articulating Paper®, Bausch Inc, Nashua, NH, USA) was photographed, in order to be available and guide the modeling of the final crown. The temporary crown remained* in situ* for a total period of two months, at the end of which it was replaced with a definitive monolithic crown in translucent zirconia (Katana®, Kuraray Noritake, Tokyo, Japan). This was obtained by modifying the design of the temporary in the previous CAD scene, taking care to adapt the cement spaces to the needs of the new material (zirconia) and to check and modify the occlusal contact points, based on the indications collected at the time of positioning of the PMMA crown. In the digital treatment (*test*), no physical models of the jaws were prepared.

The analog treatment (*control* procedure) consisted of taking a conventional impression of the implant with polyvinyl siloxane (Elite HDPlus®, Zhermack, Badia Polesine, Italy) on a generic tray after positioning of an analog impression transfer. Alginate impression was made for the antagonist arch. These impressions were disinfected and sent by post to the laboratory where plaster models were poured, in which the position of the implant was reproduced by means of an implant analog. On this model, the technician prepared a titanium abutment, purchased by the company from the dentist and supplied for this purpose, on which a temporary resin crown was produced. At the same time, the technician proceeded to the wax-up and obtained the metal structure (coping) of the crown, through a classical technique of lost-wax casting. After activation with percussion hammer of the prepared titanium abutment, the clinician applied the temporary acrylic resin crown, checking all the occlusal contacts. After a 2-month period of temporization, the patient was recalled for a second impression in polyvinyl siloxane, in which the metal coping was positioned on the abutment and retained in the impression. Starting from this impression, the technician could stratify the ceramic over the metal structure (coping) of the restoration, and about a week later the patient was recalled for the application of the final metal-ceramic crown. Again, all occlusal contacts were carefully checked, and the restoration was polished and cemented with temporary cement.

The sequence of the digital and analog treatment procedures is summarized in Table 1.

All patients were included in a hygiene-maintenance protocol, with 3 professional hygiene sessions per year, 1 every 4 months. During this period, all the complications and adverse events that could affect the restorations in the two groups were scrupulously noted. Finally, 1 year after the definitive crown was delivered, all the patients were recalled for the final examination visit, in which an endoral radiograph of* control* was also taken, and the outcomes of the study were evaluated.

### 2.4. Outcomes of the Study

The outcomes of the present randomized controlled trial were clinical and radiographic in nature, such as the implant-crown success, the biologic and prosthetic complications encountered during the observation period, and the peri-implant marginal bone loss (PIMBL); moreover, the present study investigated patient satisfaction with and the time and cost aspect of the prosthetic treatment.

#### 2.4.1. Implant-Crown Success

Implant-crown success was the main clinical outcome of the present study. An implant-supported restoration was defined as successful if it was still functioning at the end of the study without any complication, either biological or prosthetic, during the entire follow-up and at the last control appointment, 1 year after delivery. On the other hand, if only a single complication involving the implant-supported restoration occurred, the crown was included in the group of failures. The biological complications occurring during the follow-up (peri-implant mucositis and peri-implantitis), as well as the prosthetic complications affecting the restoration (complications of a mechanical nature, such as abutment loosening or abutment fracture; technical complications, such as chipping or fracturing of the restoration) were considered the reasons for the failure of the restoration. The threshold for defining peri-implantitis was set at a probing pocket depth ≥6 mm with bleeding/suppuration on probing and evidence of peri-implant bone loss >3.0 mm [34].

#### 2.4.2. Peri-Implant Marginal Bone Loss

The radiographic outcome of the present study was peri-implant bone stability, measured as PIMBL. This outcome was measured on intraoral radiographs, comparing the peri-implant bone peaks (mesial and distal) at the time of implant placement (T1) and 1 year after delivery of the definitive crown (T2). This comparison was made as described previously in other studies [29–32]. In short, the radiographs were taken using a Rinn system for alignment with a rigid film-object X-ray source coupled to a beam-aiming device. The mesial and distal marginal bone levels of all implants were measured at T1 and T2, with the help of an ocular grid (magnification 4.5x); the reference points for these measurements were the most coronal bone-to-implant contact and the implant shoulder margin. Accordingly, the PIMBL was calculated as modification in the peri-implant bone between T1 and T2, and the mean of the mesial and distal calculations was used as the final value. In order to adjust for radiographic distortion, the radiographic length of each implant was compared with the actual (true) implant length, by means of the following equation:

(1) Rx  implant  length:True  implant  length=Rx  PIMBL:True  PIMBL

All measurements were taken by an independent calibrated observer who was not part of the treating team.

#### 2.4.3. Patient Satisfaction

The degree of satisfaction and the perception of the quality of the treatment received by patients with digital and with analog procedures were investigated by means of a visual analog score (VAS) questionnaire, based on 10 specific questions. For each of these questions, patients were asked to assign a score of 0 - 10 - 20 - 30 - 40 - 50 - 60 - 70 - 80 - 90 - 100, based on their satisfaction with the treatment received (0 = absolutely dissatisfied with the treatment; 10-20-30-40 = strongly dissatisfied with the treatment; 50 = insufficiently satisfied with the treatment; 60 = sufficiently satisfied with the treatment; 70-80-90 = very satisfied with the treatment; and 100 = fully satisfied with the treatment).

The questions were as follows:

 
*(1)  Are you satisfied with the treatment?*
  with 0 = very dissatisfied and 100 = very satisfied
 
*(2)  Did you experience discomfort during the impression-taking?*
  with 0 = high discomfort and 100 = no discomfort
 
*(3)  Did you experience gag reflex/nausea during the impression-taking?*
  with 0 = strong gag reflex/nausea, and 100 = no gag reflex/nausea
 
*(4)  How comfortable was the impression procedure?*
  with 0 = uncomfortable and 100 = comfortable
 
*(5)  Does your implant-supported restoration function well?*
  with 0 = bad function and 100 = well function
 
*(6)  Do you feel secure biting on your restoration?*
  with 0 = unsecure and 100 = secure
 
*(7)  Are you pleased with the final aesthetic result?*
  with 0 = not pleased and 100 = very much pleased
 
*(8)  Is the treatment time justified?*
  with 0 = totally not justified and 100 = completely justified
 
*(9)  Is the treatment cost justified?*
  with 0 = totally not justified (too high), and 100 = completely justified
 
*(10)  Would you repeat this treatment again, if necessary?*
  with 0= absolutely not and 100 = yes, of course.

#### 2.4.4. Treatment Time

The overall treatment time and the active working time (i.e., the effective working time, excluding machine time) required for the prosthetic restoration of 1 single implant, with both treatments (digital versus analog procedure) were calculated as follows. For each treatment, the time required was calculated for the following procedures: impressions; preparation of the elements (abutment and crown) required for the provisionalization; delivery of the provisional restoration; and preparation and delivery of the final restoration. The treatment time was calculated in minutes (min).

#### 2.4.5. Cost of the Treatment

In order to assess the cost of both treatments (digital versus analog procedure) for the dentist, all the expenses related to the purchase of materials and the services of the dental laboratory were examined. The prices taken into consideration here were the official prices for the implant system used in this study, in Italy; therefore, being inclusive of value-added tax (VAT) (4% in the case of intraoral components; 22% in the case of components for extraoral use), laboratory prices reflected those of a mid-level laboratory located in northern Italy and were VAT-exempted. In this evaluation, the hourly cost of the dental clinic was not examined. The cost of the treatment was calculated in euros (€).

### 2.5. Statistical Analysis

We summarized the demographics and other characteristics of the patients enrolled in this study, using standard descriptive statistics, and compared the two groups (digital versus analog groups) using either Student's* t*-test for independent samples, or a Chi-square test for continuous and categorical variables. Similarly, the distribution of major implant characteristics (position, location, diameter, and length) between the study groups was compared using Chi-square tests. We defined an implant-crown success in terms of absence of failure or complications (biological and/or prosthetic). In each group, we estimated the prevalence of successes and tested the null hypothesis of no difference using a Chi-square test. We estimated the average PIMBL in each group with 95% confidence interval (95% CI) and tested the difference in average bone loss using Student's* t*-test. Patient satisfaction was assessed using a VAS-like score in 10 different domains; we report on the average score in each domain and on the results of a corresponding* t*-test to compare the two groups. Treatment times were measured on a subsample of n = 5 patients per study arm. We used Student's* t*-test to assess the null hypothesis of no difference between the study groups. The costs paid by the dentist for the fabrication of an implant-supported crown, through digital and analog procedure, were directly compared by examining expenses related to the purchase of materials and the dental laboratory service. The statistical analyses were performed using SAS system software, 9.4 release.

## 3. Results

### 3.1. Patient Population and Implant Distribution

In total, 50 patients (22 males and 28 females, between 24 and 76 years of age, mean age 52.6 ± 13.4 years, median 54.5 years, 95% CI 48.9–56.3 years) were selected for inclusion in the present RCT. Among these patients, only 8 were smokers and 4 were bruxists.

Patients were randomly assigned to one of the two groups (25 patients per group).

In the* test* group (digital treatment procedure), 12 males and 13 females were allocated. The patients' ages were between 24 and 68 years (mean 51.6 ± 12.3, median 54, 95% CI 46.8–56.4). Among the patients allocated in the* test* group, 4 were smokers and 3 were bruxists.

In the* control* group (analog treatment procedure), 10 males and 15 females were allocated. The patient age was between 26 and 76 years (mean 53.6 ± 14.6, median 55, 95% CI 47.9–59.3). Among the patients allocated to the* control *group, 4 were smokers and 1 was a bruxist.

Study groups did not differ by age, gender, or prevalence of smokers or bruxists (all p-values >0.05).

Overall, with regard to the overall implant distribution, 15 implants were in the maxilla and 35 in the mandible; 22 were premolars and 28 molars. The most frequently represented implant length was 10 mm (32 implants) followed by 12 mm (11 implants); only 7 implants were 8 mm in length. The most frequently represented implant diameter was 4.1 mm (42 implants), with only 8 fixtures having a diameter of 4.8 mm.

In the* test* group (digital treatment procedure), 6 implants were in the maxilla and 19 in the mandible; with regard to the position of the fixtures, 10 were premolars and 15 were molars. The most frequently used implant diameter was 4.1 mm (25 implants), whereas only 5 implants had a diameter of 4.8 mm; the most frequent implant length was 10 mm (18 implants), followed by 12 mm (5 implants) and 8 mm (2 implants).

In the* control* group (analog treatment procedure), 9 implants were in the maxilla and 16 in the mandible; with regard to the position of the fixtures, 12 were premolars and 13 molars. Finally, 22 fixtures had a diameter of 4.1 mm and 3 fixtures had a diameter of 4.8 mm; the most frequently used implant length was 10 mm (14 implants) followed by 12 mm (6 implants) and 8 mm (5 implants).

There was no difference between study groups in terms of implant location, position, length, and diameter (all p-values > 0.05; Table 2).

### 3.2. Implant-Crown Success

No patients dropped out from the study and no implants failed, for a 100% implant survival at the last follow-up control, 1 year after the delivery of the final crowns. Overall, a limited number of biologic (3/50 implants, 6%) and prosthetic (1/50 implants, 2%) complications were reported, for an implant-crown success rate of 92% (46/50 implants did not show any complication, 1 year after the delivery of the final crown).

In the* test *group, one 56-year-old, smoking, female patient experienced a biologic complication (peri-implant mucositis, with soft-tissue inflammation, exudation, and bleeding on probing in absence of peri-implant bone loss). This patient was successfully treated with a series of professional oral hygiene sessions and the problem was resolved. At the end of the study, the biologic complications in the* test* group amounted to 4% (1/25 crowns). With regard to prosthetic complications, the monolithic translucent zirconia crown of a 44-year-old male patient faced chipping, i.e., fracture of a portion of the mesio-vestibular cusp of a mandibular molar. The patient was a bruxist. The damaged crown was therefore removed and designed again from the previous intraoral scan, taking care to reduce the occlusal contacts at that critical point. The new crown was milled and delivered to the patient, and no further prosthetic complications were registered. Accordingly, at the end of the study, the prosthetic complications in the* test* group amounted to 4% (1/25 crowns). The implant-crown success in the* test* group amounted to 92% (23/25 implants without complications).

In the* control* group, two implants (1 in a 62-year-old smoking patient and 1 in a 72-year-old nonsmoking patient) were affected by peri-implant mucositis with soft-tissue inflammation and swelling, without evidence of peri-implant bone loss. Both these patients had a low level of compliance and insufficient oral hygiene. However, both these implants were successfully treated with professional oral hygiene sessions and no further problems were evidenced in these patients. At the end of the study, the incidence of biologic complications in the* control* group was 8% (2/25 implants). Conversely, no prosthetic complications were reported in this group, for an overall implant-crown success of 92% (23/25 implants without complications).

The* test* and the* control* groups did not differ in terms of complications or success probability (all p-values > 0.05; Table 2).

Examples of the digital and analog workflow are provided in Figures 2–13.

### 3.3. Peri-Implant Marginal Bone Loss

Overall, the evaluation of bone loss around the implants revealed a mean PIMBL of 0.47 ± 0.31 mm (median 0.4 mm; 95% CI 0.39–0.55 mm) after 1 year from the delivery of the final crown. In the* test *group (digital treatment procedure), the mean PIMBL amounted to 0.39 ± 0.29 mm (median 0.4 mm; 95% CI 0.28–0.5 mm). In the* control* group (analog treatment procedure), the mean PIMBL amounted to 0.54 ± 0.32 mm (median 0.6; 95% CI 0.42–0.66 mm). The average difference of -0.16 mm (95% CI: -0.33, 0.02 mm) in favor of the* test* group was not statistically significant (p = 0.08). All details on the PIMBL are summarized in Table 3.

### 3.4. Patient Satisfaction

The patient satisfaction for each treatment (digital and analog procedure) is summarized in Table 4. During the impression-taking, the digital group reported less discomfort (average difference in VAS score: +24, p < 0.0001) and were less likely to experience gag reflex/nausea (+13.2, p = 0.007), resulting in a higher rating of overall comfort during the impression procedure (+28.4, p < 0.001). Conversely, there was no difference between the study groups in the restoration phase. In addition, the digital implant received higher scores in terms of treatment time (average difference in VAS: +16, p < 0.0001) and costs (+9.2, p = 0.01). Satisfaction over the aesthetic result did not differ, with both methods scoring above 90. The overall patient satisfaction did not differ (average difference in VAS score: +2.8, p = 0.35), but only the digital group had an average score above 90. Finally, a statistically significant difference was found in the overall mean VAS (+ 9.9, p < 0.0001).

### 3.5. Treatment Time

The time required for each treatment (digital and analog procedure) is summarized in Table 5. On average, time for impression-taking was almost halved in the digital compared with the analog groups (20 ± 5 min versus 50 ± 7 min; p< 0 .0001). Overall, production times of both the temporary (600 ± 45 min versus 340 ± 37 min; p = 0.0001) and the final (525 ± 39 min versus 260 ± 26 min; p < 0.0001) crown were larger for the former group. However, when calculating active working time for the dental technician, the workflow in the* test* group was more efficient than that of the* control* group, for both the provisional (70 ± 15 min versus 340 ± 37 min; p < 0.0001) and the final crown (29 ± 9 min versus 260 ± 26 min; p < 0.0001). We observed no difference in delivery times.

### 3.6. Cost of the Treatment

The costs of the digital and analog treatment procedures were as summarized in Table 6.

For the* test* group (digital procedure), the cost of the prosthetic components amounted to €107.3 (scanbody €36.6, titanium base €70.7). The dental laboratory costs amounted to €60 for the CAD/CAM procedures for the fabrication of the provisional PMMA crown and the upper portion of the abutment (zirconia) and the assembly of the individual hybrid abutment. The CAD/CAM procedures for the fabrication of the final translucent zirconia crown cost €110. Overall, the expense for the fabrication of a single implant crown via the full digital procedure (without any model) amounted to €277.3.

For the* control* group (analog procedure), the cost of the prosthetic components amounted to €107.2 (transfer €25.0, abutment €70.7, and implant analog €11.5). The dental laboratory costs amounted to €75 for the procedures needed for the preparation of the provisional resin crown and €210 for the procedures required for the fabrication of the final metal-ceramic crown. The expense for the fabrication of a single implant crown via the conventional analog procedure (with a plaster model) amounted to €392.2.

Overall, the global cost for the fabrication of the 25 crowns of the* test* group via the digital procedure amounted to €6932.5; the global cost for the fabrication of the 25 crowns of the* control* group via the analogic procedure amounted to €9805.

## 4. Discussion

Modern digital techniques today are proliferating in dentistry, especially in implant prosthodontics [5]. The intraoral scan, in particular, gathers great consensus among dentists, because it allows one to eliminate the conventional physical impression with impression trays and materials (polyvinyl siloxane/polyether), which can be technically difficult in implant dentistry and has always represented a moment of stress for the patient [1–3, 12–14, 35]. Further advantages of the optical impression are represented by the rationalization of processes and interactions with the dental laboratory [5, 15–18]. Once the intraoral scan has been taken, the CAD/CAM procedures allow one to design and produce, by milling, individual prosthetic abutments and temporary and definitive prostheses [1–3, 5, 12–14, 16–18]. Such prostheses are clinically precise, as demonstrated by the literature in various applications [15–18, 24–28] and are fabricated of highly aesthetic materials, able to integrate perfectly into the patient's oral cavity, such as lithium disilicate [16, 23–26, 36] and zirconia [15, 17, 18, 27, 28, 37].

Despite the fact that digital procedures are conquering the market, to date only few clinical studies have compared the results obtained in implant prosthodontics with those obtained by modern digital techniques (intraoral digital impressions by means of an intraoral scanner) and conventional analog techniques (such as the classic physical impression with trays and materials, for the manufacture of metal-ceramic prostheses) [5, 15–18].

In a prospective clinical study, Joda et al. [15] evaluated the time efficiency of digital versus conventional workflows for the fabrication of single, implant-supported crowns in posterior sites. Twenty patients were selected and enrolled in the study, each one receiving a customized CAD/CAM individual hybrid abutment with a monolithic zirconia crown (*test*, digital workflow) and a standardized titanium abutment plus a porcelain-fused-to-metal crown (*control,* conventional workflow) [15]. The primary outcome of the study was time efficiency; therefore all clinical and laboratory procedures were timed, in minutes [15]. All crowns were provided within two clinical appointments. However, the mean total production time (as sum of all the clinical and laboratory steps) was significantly different (p = 0.0001): in fact, the digital workflow took a mean time of 185.4 ± 17.9 minutes, whereas the analog workflow took 223.0 ± 26.2 minutes [15]. The detailed analysis revealed a significant reduction in the time both for the clinical procedures (27.3 ± 3.4 minutes for the digital workflow versus 33.2 ± 4.9 minutes for the conventional workflow) and for the laboratory procedures (158.1 ± 17.2 minutes for the digital workflow versus 189.8 ± 25.3 minutes for the conventional workflow) [15]. The authors concluded that the digital workflow seems to be more time-efficient than the conventional workflow [15].

Similar conclusions were reported in another randomized controlled trial by the same authors [16], who evaluated the time efficiency of the single implant restoration with monolithic lithium disilicate crowns (*test*) versus porcelain fused to zirconium dioxide (*control*), in a digital workflow. Twenty patients in need of single-tooth replacement in posterior regions were randomly assigned to be restored with monolithic CAD/CAM lithium disilicate crowns bonded to prefabricated titanium abutments (*test *group, 10 patients) or to receive CAD/CAM-fabricated zirconia suprastructures and hand-layered ceramic veneering (*control* group, 10 patients) [16]. In the* test* group, no physical models were needed; in the* control* group, conversely, milled master models were produced [16]. Clinical appointments were needed for both groups of patients. However, the mean total fabrication time was significantly different (p = 0.0001), with 75.3 ± 2.1 minutes for the* test* group and 156.6 ± 4.6 minutes for the* control* group [16]. A significantly shorter (p = 0.001) mean chair time for the* test* group (20.8 ± 0.3 min) was found, when compared with the* control* group (24.1 ± 1.1 min); for the laboratory, the reduction in working time was significant too (p = 0.0001) and even more evident (54.5 ± 4.9 minutes for* test* group versus 132.5 ± 8.7 minutes for* control* group) [16]. In addition, the digital workflow with monolithic single crowns resulted in an overall reduction of 30% of the laboratory costs [16]. The authors concluded that the direct fabrication of lithium disilicate monolithic crowns was more time-efficient than the fabrication of CAD/CAM-fabricated zirconia copings veneered with ceramic [16].

In another prospective cohort trial, the authors evaluated the costs and time for implant-supported single-tooth reconstructions with a digital versus a conventional workflow [17]. Twenty patients were enrolled for rehabilitation with 2 x 20 implant crowns in a crossover study; the comparison was between digital workflow (customized titanium abutment + CAD/CAM zirconia superstructures) versus conventional workflow (standardized titanium abutments plus porcelain fused metal crowns [17]. For each treatment procedure, the costs were analyzed. At the end of the study, direct treatment costs were significantly reduced (p = 0.0004) with digital workflow (CHF 1815.35) when compared with the analogic workflow (CHF 2119.65) [17]. The total laboratory costs were significantly (p = 0.003) reduced too with the digital procedures (digital workflow: CHF 941.95; conventional workflow: CHF 1245.65) [17]. The clinical productivity rate was moreover increased with the digital pathway [17].

All these studies have shown that modern digital technologies are as clinically successful as conventional (analog) techniques and can reduce both the time required for implant-prosthetic treatment and the costs associated with it [5, 15–18]. However, there is certainly a need for more scientific evidence on this topic [5].

Our present randomized controlled clinical trial aimed to compare the reliability and effectiveness of two treatment modalities for the single implant: digital versus analog procedure—this considering not only relevant clinical aspects such as complications and marginal bone resorption, but also patient satisfaction, treatment time, and costs. We therefore selected 50 patients, each treated with a single implant positioned in the posterior areas of the jaw and allocated them to two numerically identical groups: the first group (*test*, 25 patients) was treated with modern digital procedures, while the second group (*control*, the other 25) received an analog (and therefore conventional) prosthetic treatment. It is important to emphasize how, in the present study, the block randomization has determined the formation of two homogeneous groups of patients, with regard to demographics and implant distribution. In fact, study groups did not differ by age, gender, or prevalence of smokers or bruxists, nor in terms of implant location, position, length, and diameter (all p-values > 0.05).

The first finding that emerged from our study is that 1 year after the definitive crown was delivered, there were no implant failures, for a 100% survival rate in the two groups.

There were only two complications for each group (one biological and one prosthetic complication in the* test* group; two biological complications in the* control* group) for an implant-prosthetic success (i.e., survival of implant-supported restoration without any complications) of 92%. From the clinical point of view, therefore, the two groups had virtually identical survival and success rates. The only difference between the two groups (although not statistically significant) was given by the higher incidence of biological problems (8%) in the* control* group, with two patients suffering from peri-implant mucositis. These patients had poor oral hygiene compliance, but nevertheless both these implants were successfully treated with professional oral hygiene sessions and no further problems were evidenced.

No prosthetic complications occurred in the* control* group. This is not surprising; it is well known from the literature that systems with a Morse taper implant abutment connection are characterized by a very low incidence of prosthetic complications (whether mechanical or technical) in both the short and long terms [20, 29–31, 38]. In a recent retrospective clinical study on 578 patients treated with 612 Morse taper connection implants and rehabilitated single crowns, the 15-year cumulative implant-crown success rate was 94.5% (93.1% and 94.9% for the anterior and posterior crowns, respectively), with a very low incidence of prosthetic complications (1.5%) [38]. This performance is guaranteed by the absolute mechanical stability of the connection between the abutment and the implant, in the absence of relative micromovements [39], which reduces the incidence of abutment loosening even in the long term [20, 29–31, 38].

Consistently, even in the* test* group there were no mechanical complications. However, there was a complication of a technical nature. In fact, a monolithic crown in translucent zirconia underwent chipping, with fracture of a portion of the mesiovestibular cusp of a mandibular molar. In this case, the patient was a bruxist, and the restoration was therefore replaced with a new monolithic crown, always made in CAD/CAM, but suitably modified in terms of design, in order to prevent any further chipping. In a case of technical problems of this type, the digital procedure certainly has the advantage of being able to resume and modify the original CAD design of the crown and proceed with rapid milling without having to repeat the impression; this saves a lot of time. However, the removal of a well-cemented zirconia crown can be difficult, especially if definitive cements are used. To prevent this problem, and to be able to remove the crowns if necessary, in our present study, we cemented all crowns with a temporary cement.

In our present study, there was no statistically significant difference in marginal bone resorption at 1 year from the delivery of the definitive crown, in either group; the stability of the mesial and distal bone levels at the implant was indeed optimal. In the* test* group, the mean PIMBL amounted to 0.39 ± 0.29 mm (median 0.4 mm; 95% CI 0.28–0.5 mm); in the* control* group (analog treatment procedure), the mean PIMBL amounted to 0.54 ± 0.32 mm (median 0.6; 95% CI 0.42–0.66 mm).

Since clinical and radiographic data do not show significant differences between the test and control groups, attention must be paid to patient satisfaction and the time and costs of the two different workflows.

In particular, the analysis of patient preferences showed that patients prefer the optical impression to the conventional impression, as unequivocally demonstrated in the literature [3, 13, 14, 35]. In fact, during the impression-taking, the digital group reported less discomfort (average difference in VAS score: +24, p < 0.0001) and were less likely to experience gag reflex/nausea (+13.2, p = 0.007), resulting in a higher rating of overall comfort during the impression procedure (+28.4, p < 0.001). On the other hand, there were no statistically significant differences in the patients' attitude towards the subsequent phases of treatment (i.e., the application of provisional and final restorations). However, the digital procedure received higher scores in terms of treatment time (average difference in VAS: +16, p < 0.0001) and costs (+9.2, p = 0.01); although not statistically significant, the overall satisfaction for the treatment was higher in the* test *group.

On average, time for impression-taking was almost halved in the digital compared with the analog group (20 ± 5 min versus 50 ± 7 min; p < 0.0001). Overall, production times of both the temporary (600 ± 45 min versus 340 ± 37 min; p = 0.0001) and the final (525 ± 39 min versus 260 ± 26 min; p < 0.0001) crown were larger for the former group. The latter results depended on the long sintering times of zirconia, in the* test *group. During the milling and sintering of the zirconia in the oven, however, the technician is free to do something else and to attend to other cases, without needing to guard the oven itself. Therefore, in an evaluation of the active working time, the situation was reversed, and the workflow in the* test* group was more efficient than that of the* control* group, for both the provisional (70 ± 15 min versus 340 ± 37 min; p < 0.0001) and the final crown (29 ± 9 min versus 260 ± 26 min; p< 0 .0001).

Finally, with regard to the cost of treatment, the digital procedure presented lower costs for the dentist than the conventional one (€277.3 versus €392.2, per each crown). Many of the savings were concentrated in the laboratory procedures for fabrication of the final crown (€110 for the digital crown versus €210 for the analog crown); there were no differences in the cost of impression-taking, which amounted to about €107 across the two groups, and there was a little saving in the manufacturing of the temporary restoration (€60 digital versus €75 analog). Overall, the savings for each crown using a digital procedure amounted to €114.9; considering all 25 patients, the savings guaranteed by the digital procedure amounted to €2872.5. Obviously, it must be considered that, in the face of this saving on laboratory procedures, both the dental practice and the dental laboratory must sustain substantial investments in technology to be able to work in full digital workflow (in the specific case of this study, about €30,000 for the purchase of intraoral scanner, €6000 for the purchase of CAD software, and about €35,000 for the purchase of milling machines and zirconia sintering oven), which are not necessary for working in an analog workflow. Finally, the prices for the implant-prosthetic components and materials reported in this study refer to the Italian market and are inclusive of the Italian VAT; the costs could be different in another country. Similarly, the laboratory costs reported here are those of a medium-sized dental laboratory, and they refer to a local region (that of high Lombardy, in northern Italy on the Swiss border); laboratory prices may differ, even within the same region.

The present study has limitations (such as the low number of subjects enrolled, the limited number of crowns placed, and the short follow-up time); therefore further long-term randomized controlled trials are needed to draw more specific conclusions on the reliability of full digital procedures for the fabrication of single implant crowns.

## 5. Conclusions

In the present randomized controlled trial, both the digital and the analog workflows worked successfully in restoring single-tooth gaps with implant-supported crowns, showing high success rates (92%) and a low incidence of complications (8%), and no statistically significant differences were found in the PIMBL between the two groups (*test*: 0.39 ± 0.29 mm;* control*: 0.54 ± 0.32 mm) 1 year after delivery of the definitive crown. However, patients preferred digital impressions to the conventional ones and were globally more satisfied with the digital procedures. The digital procedures were more time-efficient. In fact, on average, time for impression-taking was almost halved in the digital compared with the analog groups; and although the provisional and definitive crown fabrication involved overall more time in the* test* than in the* control* group, most of this time was machine time (i.e., the machines were operating, without the need for a continuous control by the dental technician). Therefore, when calculating active working time for the dental technician, the workflow in the* test* group was more efficient than that of the* control* group, for both the provisional (70 ± 15 min versus 340 ± 37 min; p < 0.0001) and the final crown (29 ± 9 min versus 260 ± 26 min; p < 0.0001). Finally, the digital procedure presented lower costs for the dentist than the conventional one (€277.3 versus €392.2, per crown).

Within its inherent limitations (such as the low number of subjects enrolled, the limited number of crowns placed, and the short follow-up time), the present RCT supports the concept that the digital workflow is preferred by patients, is time-effective for the dental laboratory, and is less expensive for the dentist, when compared with the analog one. Further long-term RCTs on a larger sample of patients are required to draw more specific conclusions on the reliability and efficacy of full digital workflow for the fabrication of implant-supported single crowns.

## Figures and Tables

**Figure 2:**
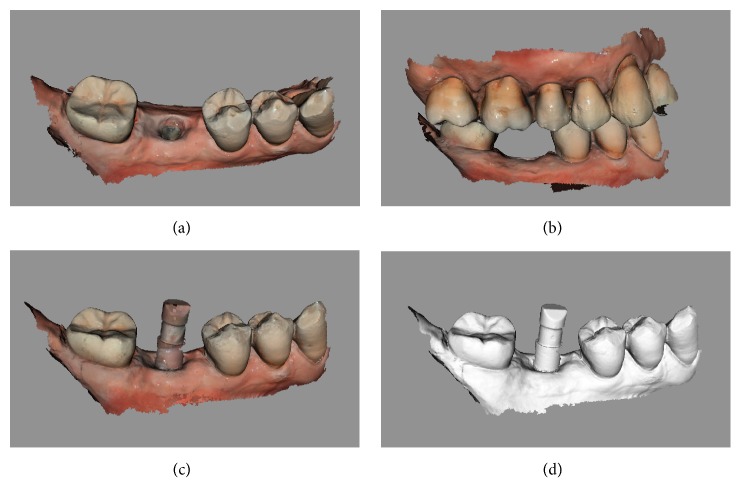
*Test* group (digital workflow). Digital impressions of a first mandibular molar with CS 3600® (Carestream Dental, Rochester, NY, USA). (a) impression of the mucosa after removal of the healing abutment,  .PLY; (b) registration of occlusion,  .PLY; (c) impression with the scanbody* in situ*;  .PLY (d)  .STL file of the impression with the implant scanbody.

**Figure 3:**
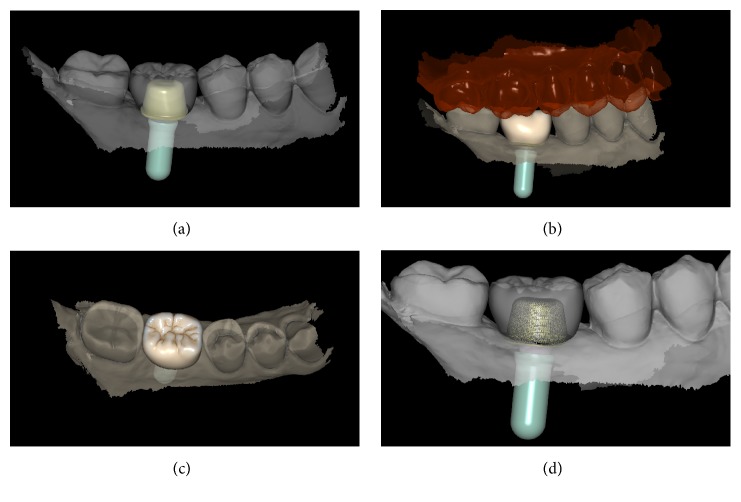
*Test* group (digital workflow). Computer-assisted design (CAD) phases for the design of the final zirconia abutment and the provisional PMMA crown with Exocad DentalCAD® (Exocad, Darmstadt, Germany). (a) The final zirconia abutment and the provisional PMMA crown have been designed; (b) occlusal contacts; (c) occlusal view of the provisional PMMA crown design; (d) relationship between the abutment and the crown using the transparency tool.

**Figure 4:**
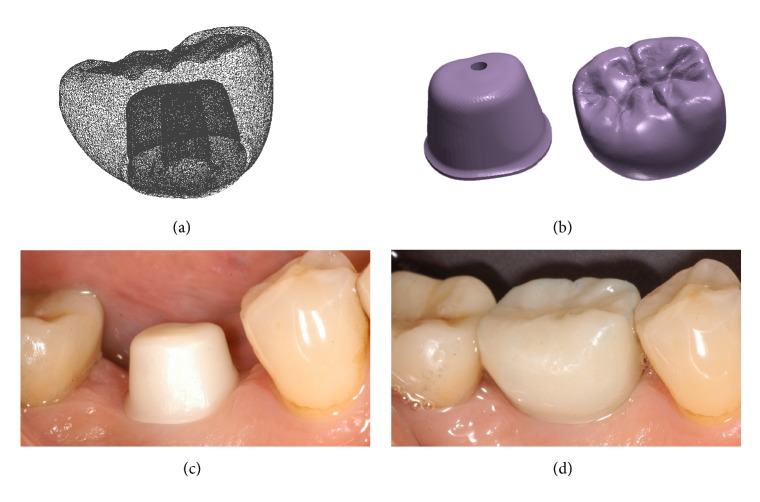
*Test* group (digital workflow). (a) The quality of the mesh is verified before milling; (b) the  .STL files are ready to be produced by the milling machines: a 5-axis milling unit is used to mill the zirconia abutment (Roland DWX-50®, Roland Easy Shape, Ascoli Piceno, Italy) whereas a 4-axis milling unit is used to mill the provisional PMMA crown (Roland DWX-4®, Roland Easy Shape, Ascoli Piceno, Italy); (c) the individual hybrid abutment is placed in position; (d) the provisional crown is positioned on the individual abutment and a careful check of the occlusal and interproximal contacts is made before characterization. The provisional crown will remain in situ for 3 months.

**Figure 5:**
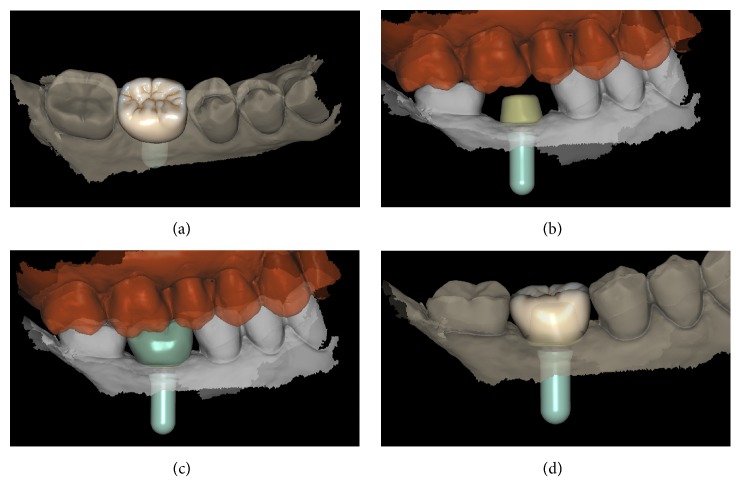
*Test* group (digital workflow). The final zirconia crown is designed in the Exocad DentalCAD® (Exocad, Darmstadt, Germany) software. (a) Occlusal view; (b) distance between the top of the individual zirconia abutment and the opposing arch; (c) lateral view of the final crown design; (d) photorealistic rendering of the final crown.

**Figure 6:**
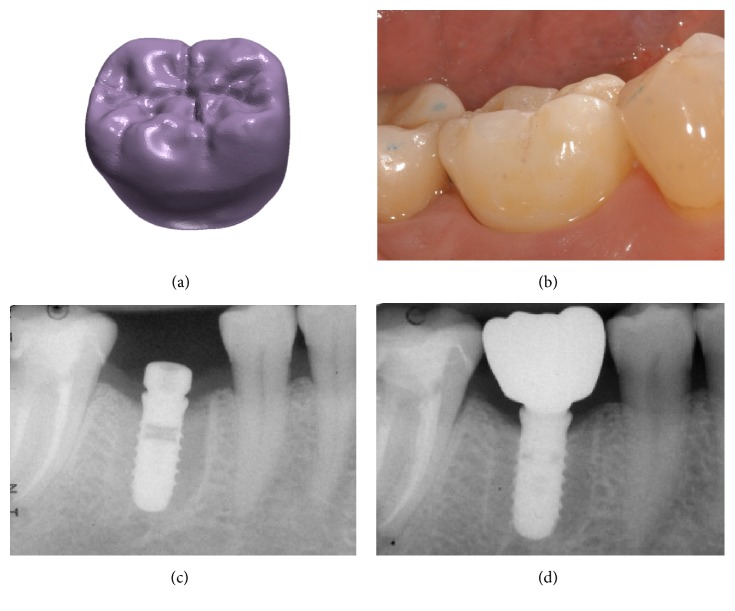
*Test* group (digital workflow). (a) the  .STL file of the final zirconia crown is ready to be milled; (b) delivery of the final monolithic zirconia crown; (c) radiograph at the time of implant placement; (d) radiograph 1 year after delivery of the definitive crown.

**Figure 7:**
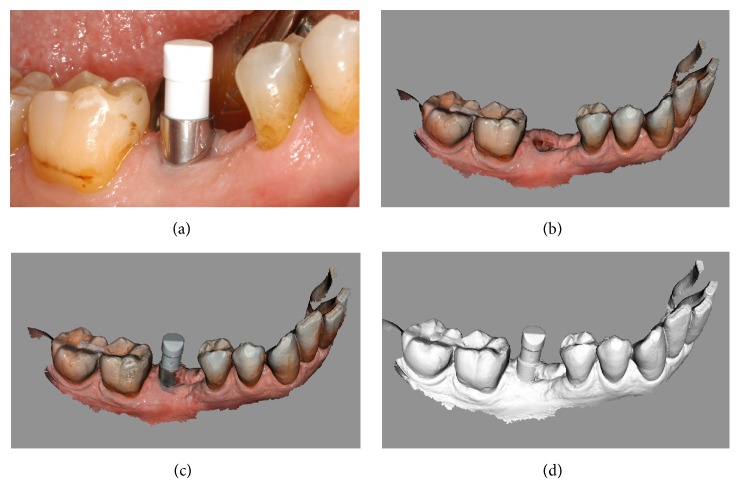
*Test* group (digital workflow). Digital impressions of a first mandibular molar with CS 3600® (Carestream Dental, Rochester, NY, USA). (a) Intraoral view of the implant scanbody; (b) impression of the mucosa after removal of the healing abutment,  .PLY; (c) impression with the scanbody* in situ*;  .PLY (d)  .STL file of the impression with the implant scanbody.

**Figure 8:**
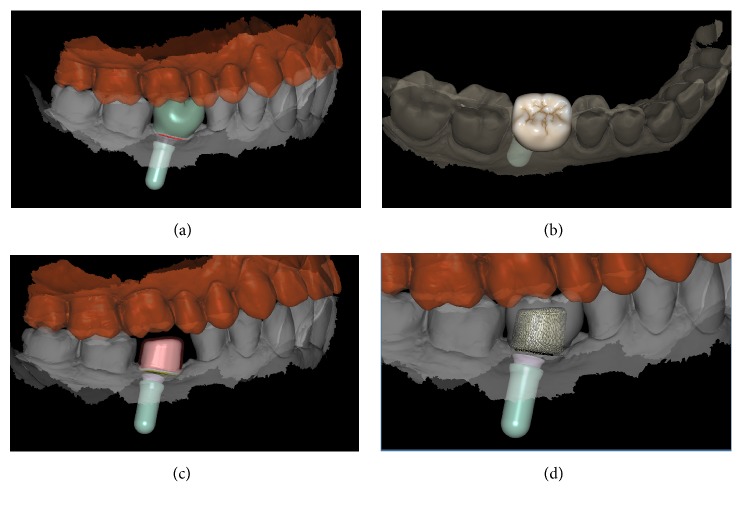
*Test* group (digital workflow). Computer-assisted design (CAD) phases for the design of the final zirconia abutment and the provisional PMMA crown with Exocad DentalCAD® (Exocad, Darmstadt, Germany). (a) The final zirconia abutment and the provisional PMMA crown have been designed; (b) occlusal view of the temporary crown; (c) space between the individual zirconia abutment and the opposing arch; (d) relationship between the abutment and the crown using the transparency tool.

**Figure 9:**
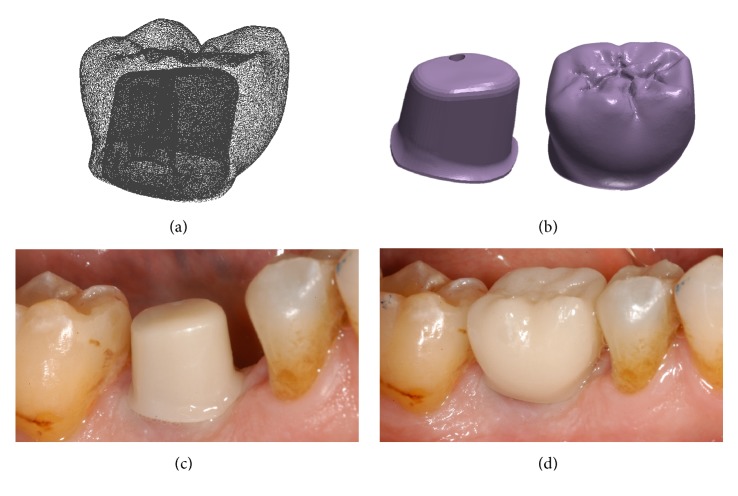
*Test* group (digital workflow). (a) The quality of the mesh is verified before milling; (b) the  .STL files are ready to be produced by the milling machines: a 5-axis milling unit is used to mill the zirconia abutment (Roland DWX-50®, Roland Easy Shape, Ascoli Piceno, Italy) whereas a 4-axis milling unit is used to mill the provisional PMMA crown (Roland DWX-4®, Roland Easy Shape, Ascoli Piceno, Italy); (c) the individual hybrid abutment is placed in position; (d) the provisional crown is positioned on the individual abutment and a careful check of the occlusal and interproximal contacts is made before characterization. The provisional crown will remain in situ for 3 months.

**Figure 10:**
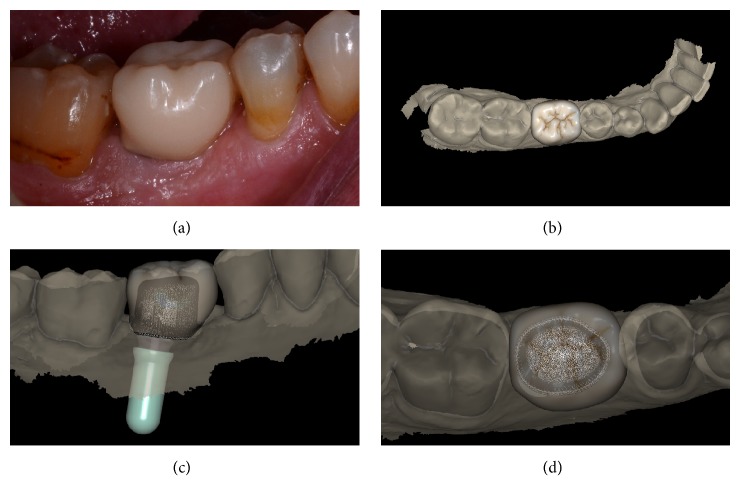
*Test* group (digital workflow). (a) The provisional PMMA crown has been in situ for 3 months and it is time to replace it with the final zirconia crown; (b) the final zirconia crown is designed in the CAD software; (c) spatial relationships between the final crown and the individual hybrid abutment, lateral view; (d) spatial relationships between the final crown and the individual hybrid abutment, occlusal view.

**Figure 11:**
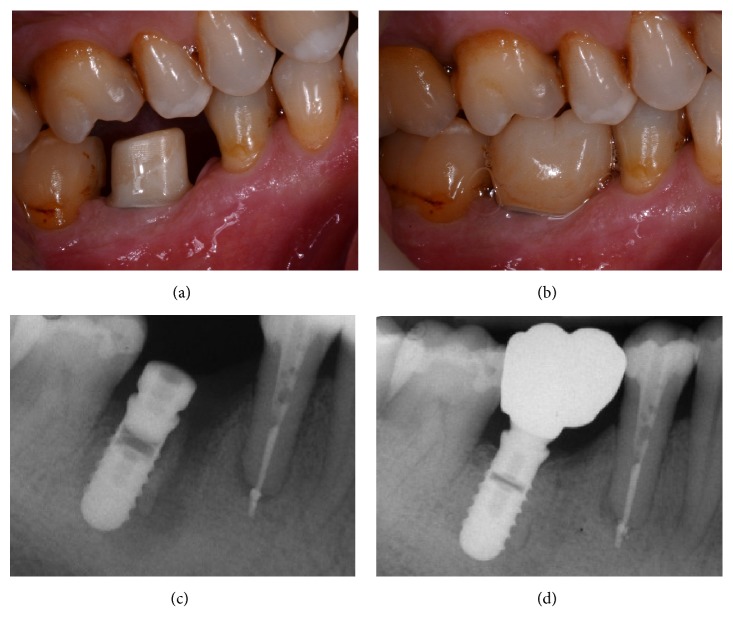
*Test* group (digital workflow). (a) Lateral view of the zirconia abutment, 3 months after placement; (b) delivery of the final monolithic zirconia crown; (c) radiograph at the time of implant placement; (d) radiograph 1 year after delivery of the definitive crown.

**Figure 12:**
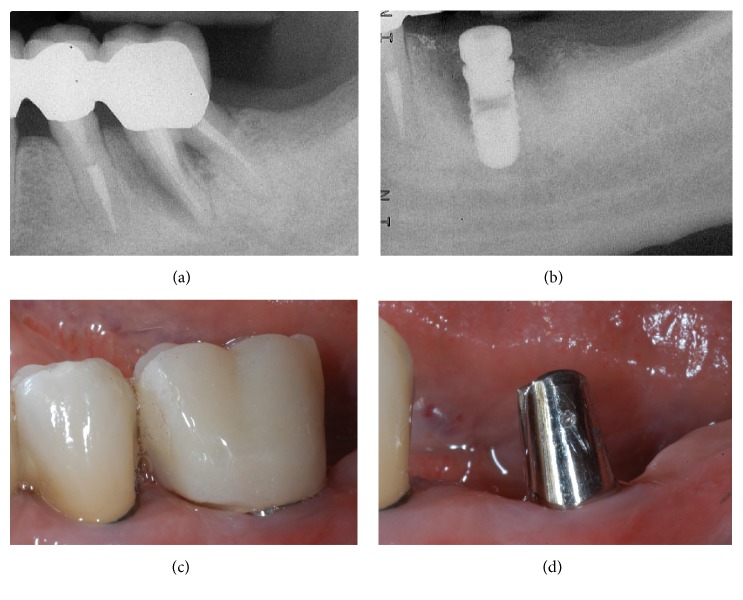
*Control* group (analog workflow). (a) Radiograph of the failing tooth; (b) radiograph of the implant at placement; (c) the provisional restoration is delivered to the patient; (d) lateral view of the titanium abutment, 3 months after placement, before the delivery of the final crown.

**Figure 13:**
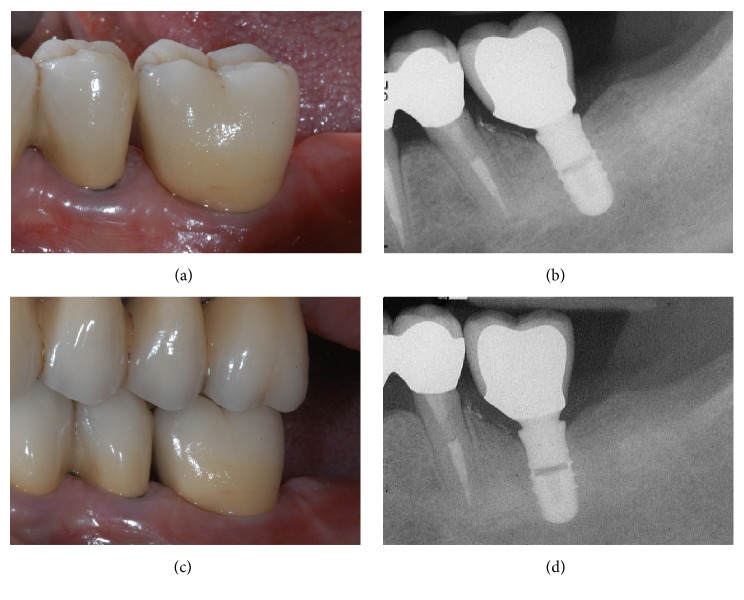
*Control* group (analog workflow). (a) Delivery of the final metal-ceramic crown; (b) radiograph of the final metal-ceramic crown at placement; (c) the metal-ceramic crown one year after placement; (d) radiograph 1 year after delivery of the definitive crown.

**Table 1:** Digital versus analog procedures: workflow.

Digital procedure	Analog procedure
First appointment in dental clinic (1) Healing abutment (HA) removal (2) Intraoral scan *(i) implant site without HA + adjacent teeth* *(ii) opposite arch* *(iii) occlusal registration* *(iv) scanbody insertion* *(v) implant site with scanbody* (3) Colour determination (4) HA re-insertion	First appointment in dental clinic: (1) Healing abutment (HA) removal (2) Impression taking *(i) implant arch in polyvinyl siloxane with transfer* *(ii) opposite arch in alginate* *(iii) occlusal registration* (3) Colour determination (4) HA re-insertion

In dental laboratory: (1) Computer assisted design (CAD) *(i) design of the upper portion of the individual abutment* *(ii) design of the provisional crown* (2) Computer assisted manufacturing (CAM) *(i) milling of the upper portion of the individual abutment in zirconia* *(ii) the zirconia abutment is sintered* *(iii) milling of the provisional crown in polymethylmethacrylate (PMMA)* *(iv) polishing and characterization of the provisional PMMA crown* (3) Assembly of the individual abutment by cementing the upper zirconia portion over the selected titanium base	In dental laboratory: (1) Preparation of the provisional resin crown *(i) the implant analog is assembled with the transfer* *(ii) plaster models are poured* *(iii) the transfer is removed* *(iv) a prefabricated titanium abutment is inserted in the implant analog* *(v) the abutment is manually prepared into the desired form* *(vi) wax-up of the provisional crown* *(vii) a silicon mask is prepared over the wax-up* *(viii) the wax is removed* *(ix) the resin is injected into the silicon mask and polymerized in order to obtain the provisional crown* *(x) the provisional crown is polished and characterized* (2) Preparation of the metal coping (i) wax-up of the coping (ii) lost-wax casting for the fabrication of the metal coping (iii) the metal coping is polished

Second appointment in dental clinic: (1) Healing abutment removal (2) Insertion and activation of the individual hybrid abutment (3) Delivery and cementation of the provisional crown	Second appointment in dental clinic (1) Healing abutment removal (2) Insertion and activation of the titanium abutment (3) Delivery and cementation of the provisional crown

In dental laboratory: (1) Computer assisted design (CAD) *(i) design of the final crown* (2) Computer assisted manufacturing (CAM) *(i) milling of the final crown in zirconia* *(ii) the zirconia crown is sintered* *(iii) the zirconia crown is characterized*	Third appointment in dental clinic: (1) The provisional crown is removed (2) The metal coping is placed on the titanium abutment (3) Impression taking *(i) implant arch in polyvinyl siloxane with the metal coping in position* *(ii) opposite arch in alginate* *(iii) occlusal registration*

Third appointment in dental clinic: (1) Removal of the provisional crown (2) Delivery and cementation of the final monolithic translucent zirconia crown	In dental laboratory: (1) Preparation of the final metal-ceramic crown *(i) plaster models are poured with coping in position* *(ii) the ceramic is stratified over the metal coping and sintered* *(iii) the ceramic is polished*

	Fourth appointment in dental clinic: (1) Removal of the provisional crown (2) Delivery and cementation of the final metal-ceramic crown

**Table 2:** Implant distribution and number of failures and complications registered in the two groups (digital versus analog procedures).

	*All implants*	*Treatment procedure*		*p-value ∗*
		*Digital*	*Analog*	
N	50	25	25	-

*Implant location*				

*Maxilla*	15/50 (30%)	6/25 (24%)	9/25 (36%)	0.35
*Mandible*	35/50 (70%)	19/25 (76%)	16/25 (64%)	

*Implant position*				

*Premolars*	22/50 (44%)	10/25 (40%)	12/25 (48%)	0.57
*Molars*	28/50 (56%)	15/25 (60%)	13/25 (52%)	

*Implant length*				

*8 mm*	7/50 (14%)	2/25 (8%)	5/25 (20%)	0.39
*10 mm*	32/50 (64%)	18/25 (72%)	14/25 (56%)	
*12 mm*	11/50 (22%)	5/25 (20%)	6/25 (24%)	

*Implant diameter*				

*4.1 mm*	42/50 (84%)	20/25 (80%)	22/25 (88%)	0.44
*4.8 mm*	8/50 (16%)	5/25 (20%)	3/25 (12%)	

*Implant failures*	0/50 (0%)	0/0 (0%)	0/0 (0%)	ne

*Biologic complications*	3/50 (6%)	1/25 (4%)	2/25 (8%)	0.55

*Prosthetic complications*	1/50 (2%)	1/25 (4%)	0/25 (0%)	0.31

*Implant-crown success*	46/50 (92%)	23/25 (92%)	23/25 (92%)	1.0

N = number of the fixtures examined.

*∗* = Chi-square test.

ne: not estimable (no observed failures).

**Table 3:** Peri-implant marginal bone loss (PIMBL), in mm, measured one year after the delivery of the final crown, in the two different groups (digital versus analog treatment).

	*All implants*	*Treatment procedure*		*p value*
		*Digital*	*Analog*	
N	50	25	25	-

*Median (25*° *-75*° * pct)*	0.4 (0.3; 0.6)	0.4 (0.2; 0.5)	0.6 (0.4; 0.7)	0.06∧

*Mean (95*%* CI)*	0.47 (0.38; 0.56)	0.39 (0.27; 0.51)	0.55 (0.41; 0.68)	0.08*∗*

N = number of the fixtures examined.

∧: Kruskal-Wallis test; *∗* = Student's *t*-test.

**Table 4:** Patient satisfaction in the two different groups (digital versus analog treatment).

	*Mean VAS*		*p value∗*
	*Digital*	*Analog*	
*(1 ) Are you satisfied with the treatment?*	90 (±10)	87.2 (±11)	0.35

*(2) Did you experience discomfort during the impression taking? *	96.8 (±4.7)	72.8 (±18.6)	<.0001

*(3) Did you experience gag reflex/nausea during the impression taking?*	97.2 (±5.4)	84 (±21.7)	0.007

*(4) How comfortable was the impression procedure? *	97.6 (±4.3)	69.2 (±13.8)	<.0001

*(5) Do your implant-supported restoration function well?*	96.4 (±4.9)	93.6 (±8.1)	0.15

*(6) Do you feel secure biting on your restoration?*	92.8 (±7.9)	92.4 (±8.3)	0.86

*(7) Are you pleased with the final aesthetic result?*	93.2 (±8.0)	92 (±7.6)	0.59

*(8) Is the treatment time justified?*	97.2 (±7.3)	81.2 (±11.3)	<.0001

*(9) is the treatment cost justified?*	82.4 (±13)	73.2 (±11.8)	0.01

*(10) Would you repeat this treatment, if necessary?*	91.2 (±8.8)	90.4 (±7.3)	0.73

*Overall mean VAS*	93.5 (±3.3)	83.6 (±4.0)	<.0001

*∗* = Student's *t*-test for independent samples, using Satterthwaite method in case of unequal group variances.

**Table 5:** Treatment time, in minutes (min), for the two different groups (digital versus analog treatment).

	*Treatment procedure*		*p value∗*
	*Digital*	*Analog*	
*Impression taking∗∗*	(i) HA removal (1 min) (ii) Intraoral scan (15 min) (iii) Colour determination (3 min) (iv) HA re-insertion (1 min) Total = 20 (± 5) min Active working time = 20 (± 5) min	(first impression) (i) HA removal (1 min) (ii) Impression taking (20 min) (iii) Colour determination (3 min) (iv) HA re-insertion (1 min) Total = 25 (± 5) min (second impression) (i) resin crown removal (1 min) (ii) Metal coping placement (1 min) (iii) Impression taking (20 min) (iv) resin crown re-cementation (3 min) Total = 25 (± 4) min Active working time= 50 min (± 7)	<.0001

*Production of the temporary crown*	(i) CAD of the individual abutment (30 min) (ii) CAD of the crown (25 min) (iii) Milling of the zirconia abutment (25 min) (iv) Sinterization of the zirconia abutment (480 min) (v) Milling of the PMMA crown (25 min) (vi) Polishing and characterization of the PMMA crown (5 min) (vii) Assembly of the individual hybrid abutment (10 min) Total = 600 min (± 45) Active working time = 70 min (± 15 min)	(i) Preparation of the temporary resin crown (120 min) (ii) Polishing and characterization of the temporary resin crown (20 min) (iii) Preparation of the metal coping (180 min) (iv) polishing of the metal coping (20 min) Total = 340 min (± 37) Active working time = 340 min (± 37)	0.0001

*Delivery of the temporary crown*	(i) HA removal (1 min) (ii) Insertion and activation of the individual hybrid abutment (5 min) (iii) Delivery and cementation of the temporary crown (5 min) Total = 11 min (± 5) Active working time = 11 min (± 5)	(i) HA removal (1 min) (ii) Insertion and activation of the titanium abutment (3 min) 3. Delivery and cementation of the temporary crown (5 min) Total = 9 min (± 4) Active working time = 9 min (± 4)	0.5

*Preparation of the final crown*	(i) CAD of the final crown (10 min) (ii) milling of the final translucent zirconia crown (25 min) (iii) sinterization of the final translucent zirconia crown (480 min) (iv)characterization of the final translucent zirconia crown (10 min) Total = 525 min (± 39) Active working time = 25 min (± 9 min)	(i) Preparation of the metal-ceramic crown (240 min) (ii) Polishing of the metal-ceramic crown (20 min) Total = 260 min (± 26) Active working time = 260 min (± 26)	<0.0001

*Delivery of the final crown*	(i) Removal of the temporary crown (2 min) (ii) Placement, adjustments, and final cementation of the monolithic translucent zirconia crown (12 min) Total = 14 min (± 5) Active working time = 14 min (± 5)	(i) Removal of the temporary crown (2 min) (ii) Placement, adjustments, and final cementation of the metal-ceramic crown (10 min) Total = 12 min (± 5) Active working time = 12 min (± 5)	0.5

*∗* = Student's *t*-test for independent samples.

*∗∗* for the analog procedure, two impressions are regularly taken, one with the transfer and the other with the metal coping in position.

**Table 6:** Costs for materials and dental laboratory procedures, in euro (€), in the two different groups (digital versus analog treatment).

	*Treatment procedure*	
	*Digital*	*Analog*
*Materials *	Scanbody (€ 36.6) Abutment (€ 70.7) Total = € 107.3	Transfer (€ 25.0) Abutment (€ 70.7) Implant analog (€ 11.5) Total = € 107.2 euro

*Laboratory for production of the temporary crown*	Hybrid abutment (€ 50) PMMA crown (€ 10) Total = € 60 euro	Abutment preparation (€ 50) Resin crown (€ 25) Total = € 75

*Laboratory for production of the final crown*	Zirconia crown (€ 80) Characterization (€ 30) Total = € 110	Metal-ceramic crown (€ 210) Total = € 210

*Overall*	€ 277,3	€ 392,2

## Data Availability

The data used to support the findings of this study are available from the corresponding author upon reasonable request.
